# Supplementary material for: Genome-Wide Identification of Aquaporin Genes in Adzuki Bean (Vigna angularis) and Expression Analysis under Drought Stress
Source: Int J Mol Sci. 2022 Dec 19;23(24):16189. doi: 10.3390/ijms232416189 (PMC9782098; doi:10.3390/ijms232416189)
Supplement: Supplementary file 1 [file ijms-23-16189-s001.zip › ijms-2011985-supplementary.pdf]

Table S1. List of primers used for measuring *V. angularis* aquaporin expression by RT–qPCR.

Gene Name	Primer sequence 5′– > 3′ (Forward/Reverse Primer))	Amplicon Size (bp)
VaPIP1-1	F: GCCCAACGTGTACGAGAG<br>R: CACGAAGGTGCCGATGAT	100
VaPIP1-7	F: CTGTTCCTGTACATCACCATCC<br>R: AAGATCATGCCGCCGAAG	110
VaPIP2-1_	F: TGACCCAGTGGAGCTTCTA<br>R: GGCTCTTGTAGCCGATCAC	99
VaPIP2-5	F: CCAGAAGAGCTACTACAACAGG<br>R: CACGAAGGTGCCGATGAT	103
VaACTINE	F: CTAAGGCTAATCGTGAGAA<br>R: CGTAAATAGGAACCGTGT	100

Table S2. NCBI-CDD search results: Summary details of the conserved motifs identified and user queries added to the CD multiple sequence alignment.

Query	Chromosome	Type	Title	Coordinates	Complete Size	Mapped Size	Source Domain
>VaTIP2-2	-	specific	Asn-Pro-Ala signature motifs	N83,P84,A85,N197,P198,A199	6	6	238,204
		specific	amphipathic channel	H63,H81,V82,N83,G193,G194,N197,R200	8	8	238,204
>VaTIP4-1	-	specific	Asn-Pro-Ala signature motifs	N79,P80,A81,N193,P194,A195	6	6	238,204
		specific	amphipathic channel	A59,H77,L78,N79,A189,A190,N193,R196	8	8	238,204
>VaTIP1-3	-	specific	Asn-Pro-Ala signature motifs	N85,P86,A87,N198,P199,A200	6	6	238,204
		specific	amphipathic channel	H65,H83,V84,N85,G194,A195,N198,V201	8	8	238,204
>VaNIP1-3	Chr 1	specific	Asn-Pro-Ala signature motifs	N97,P98,A99,N209,P210,V211	6	6	238,204
		specific	amphipathic channel	W77,H95,F96,N97,G205,A206,N209,R212	8	8	238,204
>VaNIP1-2	Chr 1	specific	Asn-Pro-Ala signature motifs	N95,P96,A97,N207,P208,A209	6	6	238,204
		specific	amphipathic channel	W75,H93,F94,N95,G203,A204,N207,R210	8	8	238,204
>VaNIP4-1	Chr 2	generic	Asn-Pro-Ala signature motifs	N120,P121,A122,N231,P232,A233	6	6	238,204
		generic	amphipathic channel	A100,H118,V119,N120,G227,G228,N231,R234	8	8	238,204
>VaNIP3-1	Chr 2	specific	Asn-Pro-Ala signature motifs	N132,P133,S134,N243,P244,V245	6	6	238,204
		specific	amphipathic channel	H130,L131,N132,G239,G240,N243,R246	8	7	238,204
>VaPIP2-3	Chr 2	specific	Asn-Pro-Ala signature motifs	N107,P108,A109,N228,P229,A230	6	6	238,204
		specific	amphipathic channel	F87,H105,I106,N107,G224,T225,N228,R231	8	8	238,204
>VaTIP3-2	Chr 2	specific	Asn-Pro-Ala signature motifs	N85,P86,A87,N199,P200,A201	6	6	238,204
		specific	amphipathic channel	H65,H83,V84,N85,G195,A196,N199,R202	8	8	238,204
>VaPIP1-2	Chr 3	specific	Asn-Pro-Ala signature motifs	N116,P117,A118,N237,P238,A239	6	6	238,204
		specific	amphipathic channel	F96,H114,I115,N116,G233,T234,N237,R240	8	8	238,204
>VaPIP1-3	-	Non specific	-	-	-	-	-
>VaPIP1-4	Chr 3	Non specific	-	-	-	-	-
>VaSIP2-1	Chr 3	generic	Asn-Pro-Ala signature motifs	N68,P69,L70,N179,P180,A181	6	6	238,204

		generic	amphipathic channel	S48,A66,Y67,N68,G175,G176,N179,S182	8	8	238,204
>VaNIP2-1	Chr 3	specific	Asn-Pro-Ala signature motifs	N105,P106,A107,N216,P217,A218	6	6	238,204
		specific	amphipathic channel	A85,H103,M104,N105,G212,G213,N216,R219	8	8	238,204
>VaPIP2-4	Chr 3	specific	Asn-Pro-Ala signature motifs	N104,P105,A106,N225,P226,A227	6	6	238,204
		specific	amphipathic channel	F84,H102,I103,N104,G221,T222,N225,R228	8	8	238,204
>VaPIP1-7	Chr 3	specific	Asn-Pro-Ala signature motifs	N117,P118,A119,N239,P240,A241	6	6	238,204
		specific	amphipathic channel	F97,H115,I116,N117,G235,T236,N239,R242	8	8	238,204
>VaTIP1-2	Chr 5	specific	Asn-Pro-Ala signature motifs	N85,P86,A87,N199,P200,A201	6	6	238,204
		specific	amphipathic channel	H65,H83,V84,N85,G195,A196,N199,V202	8	8	238,204
>VaNIP3-2	Chr 5	specific	Asn-Pro-Ala signature motifs	N134,P135,A136,N245,P246,V247	6	6	238,204
		specific	amphipathic channel	T114,H132,L133,N134,G241,G242,N245,R248	8	8	238,204
>VaNIP3-3	-	Non specific	-	-	-	-	-
>VaTIP2-1	Chr 5	specific	Asn-Pro-Ala signature motifs	N83,P84,A85,N196,P197,A198	6	6	238,204
		specific	amphipathic channel	H63,H81,V82,N83,G192,G193,N196,R199	8	8	238,204
>VaPIP2-5	Chr 5	specific	Asn-Pro-Ala signature motifs	N109,P110,A111,N230,P231,A232	6	6	238,204
		specific	amphipathic channel	F89,H107,I108,N109,G226,T227,N230,R233	8	8	238,204
>VaTIP2-3	Chr 6	specific	Asn-Pro-Ala signature motifs	N83,P84,A85,N196,P197,A198	6	6	238,204
		specific	amphipathic channel	H63,H81,L82,N83,G192,G193,N196,R199	8	8	238,204
>VaTIP1-1	Chr 7	specific	Asn-Pro-Ala signature motifs	N85,P86,A87,N199,P200,A201	6	6	238,204
		specific	amphipathic channel	H65,H83,V84,N85,G195,A196,N199,V202	8	8	238,204
>VaPIP1-6	Chr 7	specific	Asn-Pro-Ala signature motifs	N117,P118,A119,N239,P240,A241	6	6	238,204
		specific	amphipathic channel	F97,H115,I116,N117,G235,T236,N239,R242	8	8	238,204
>VaXIP1-1	Chr 8	generic	Asn-Pro-Ala signature motifs	N100,P101,I102,N232,P233,A234	6	6	238,204
		generic	amphipathic channel	V80,H98,M99,N100,G228,A229,N232,R235	8	8	238,204
>VaPIP2-7	Chr 8	specific	Asn-Pro-Ala signature motifs	N104,P105,A106,N225,P226,A227	6	6	238,204
		specific	amphipathic channel	F84,H102,I103,N104,G221,T222,N225,R228	8	8	238,204

>VaXIP2-1	Chr 8	generic	Asn-Pro-Ala signature motifs	S62,P63,V64,N194,P195,A196	6	6	238,204
		generic	amphipathic channel	V38,H60,M61,S62,G190,V191,N194,R197	8	8	238,204
>VaTIP5-1	Chr 8	generic	Asn-Pro-Ala signature motifs	N86,P87,A88,N199,P200,A201	6	6	238,204
		generic	amphipathic channel	S66,H84,V85,N86,G195,G196,N199,C202	8	8	238,204
>VaPIP2-8	Chr 8	specific	Asn-Pro-Ala signature motifs	N104,P105,A106,N225,P226,A227	6	6	238,204
		specific	amphipathic channel	F84,H102,I103,N104,G221,T222,N225,R228	8	8	238,204
>VaPIP2-6	Chr 8	specific	Asn-Pro-Ala signature motifs	N107,P108,A109,N228,P229,A230	6	6	238,204
		specific	amphipathic channel	F87,H105,I106,N107,G224,T225,N228,R231	8	8	238,204
>VaNIP1-4	Chr 9	generic	Asn-Pro-Ala signature motifs	N82,P83,G84,N195,P196,A197	6	6	238,204
		generic	amphipathic channel	W62,H80,F81,N82,G191,A192,N195,R198	8	8	238,204
>VaNIP1-5	Chr 7	Non specific	-	-	-	-	-
>VaNIP1-1	Chr 9	specific	Asn-Pro-Ala signature motifs	N100,P101,A102,N212,P213,A214	6	6	238,204
		specific	amphipathic channel	W80,H98,F99,N100,G208,A209,N212,R215	8	8	238,204
>VaSIP1-1	Chr 3	Non specific	-	-	-	-	-
>VaSIP1-2	Chr 10	generic	Asn-Pro-Ala signature motifs	N78,P79,N82,N193,P194,A195	6	6	238,204
		generic	amphipathic channel	S76,F77,N78,G189,P190,N193,F196	8	7	238,204
>VaPIP2-1	Chr 10	specific	Asn-Pro-Ala signature motifs	N105,P106,A107,N226,P227,A228	6	6	238,204
		specific	amphipathic channel	F85,H103,I104,N105,G222,T223,N226,R229	8	8	238,204
>VaTIP3-1	Chr 10	specific	Asn-Pro-Ala signature motifs	N85,P86,A87,N199,P200,A201	6	6	238,204
		specific	amphipathic channel	H65,H83,V84,N85,G195,A196,N199,L202	8	8	238,204
>VaPIP2-2	Chr 10	specific	Asn-Pro-Ala signature motifs	N105,P106,A107,N226,P227,A228	6	6	238,204
		specific	amphipathic channel	F85,H103,I104,N105,G222,T223,N226,R229	8	8	238,204
>VaPIP1-1	Chr 11	specific	Asn-Pro-Ala signature motifs	N116,P117,A118,N237,P238,A239	6	6	238,204
		specific	amphipathic channel	F96,H114,I115,N116,G233,T234,N237,R240	8	8	238,204
>VaPIP1-5	Chr 11	specific	Asn-Pro-Ala signature motifs	N114,P115,A116,N236,P237,A238	6	6	238,204
		specific	amphipathic channel	F94,H112,I113,N114,G232,T233,N236,R239	8	8	238,204

Note: Complete size, total number of residues in the conserved feature/site that has been annotated on the domain model; Mapped size, the number of

residues in the query protein sequence that match residues in the conserved feature/site that was annotated on the domain model.
